# Clinical Utility of Intraoperative Parathyroid Hormone Measurement in Children and Adolescents Undergoing Total Thyroidectomy

**DOI:** 10.3389/fendo.2019.00760

**Published:** 2019-11-06

**Authors:** Steven D. Tsai, Sogol Mostoufi-Moab, Samantha Bauer, Ken Kazahaya, Colin P. Hawkes, N. Scott Adzick, Andrew J. Bauer

**Affiliations:** ^1^Perelman School of Medicine, University of Pennsylvania, Philadelphia, PA, United States; ^2^Division of Pediatric Oncology, Children's Hospital of Philadelphia, Philadelphia, PA, United States; ^3^Division of Endocrinology and Diabetes, Children's Hospital of Philadelphia, Philadelphia, PA, United States; ^4^Orthopaedic Institute for Children, Los Angeles, CA, United States; ^5^Division of Pediatric Otolaryngology, Children's Hospital of Philadelphia, Philadelphia, PA, United States; ^6^Division of Pediatric General, Thoracic, and Fetal Surgery, Children's Hospital of Philadelphia, Philadelphia, PA, United States

**Keywords:** parathyroid hormone, pediatric, pediatric thyroidectomy, intraoperative PTH, hypocalcemia, recovery time

## Abstract

**Background:** Hypoparathyroidism is one of the most common complications for patients undergoing total thyroidectomy. Our study's primary objective was to assess if intraoperative PTH levels correlate with parathyroid gland function recovery time in pediatric patients following total thyroidectomy.

**Methods:** Retrospective review of pediatric patients who underwent thyroid surgery at CHOP for demographics and laboratory test values (calcium, phosphorus, and parathyroid hormone). We defined Time of Recovery (TOR) as the time difference from first intra-operative parathyroid hormone level (ioPTH) timepoint until normalization of PTH (> 10 pg/mL) post-thyroidectomy. Calcium and vitamin D supplements were weaned following normalization of calcium and phosphorous levels postoperatively. Patients were excluded if they lacked three intraoperative PTH timepoints or were missing postoperative follow-up PTH data.

**Results:** 65 patients (54 female), median age 15 (range 5–23 years), underwent thyroid surgery and met study inclusion criteria. The correlations of 2nd and 3rd ioPTHs with TOR were statistically significant (*p* < 0.05): the lower the ioPTH, the greater the recovery time. Stratifying patients into high-risk (2nd ioPTH ≤ 10 pg/mL), moderate-risk (2nd ioPTH between 10 and 20 pg/mL), and low-risk (2nd ioPTH ≥ 20 pg/mL) tertiles, the TOR decreased by orders of magnitudes from an average of 43.13 ± 76.00 to 6.10 ± 17.44 to 1.85 ± 6.20 days. These differences were statistically significant (*p* < 0.05).

**Conclusions:** Our study results confirm the usefulness of intraoperative PTH levels to predict pediatric patient recovery post-surgery and provides useful anticipatory guidance to optimize timing and frequency of postoperative laboratory surveillance.

## Introduction

Postoperative hypoparathyroidism is one of the most commonly reported surgical complications associated with thyroid surgery in children and adults (1, 2). The incidence of this complication varies widely in the literature and directly correlates with total thyroidectomy and repeat thyroid surgery, and inversely correlates with the surgical volume of the surgeon and patient age (3–6). In a recent review of 467 thyroidectomy patients treated between 2009 and 2017, we reported a 0.6% rate of permanent hypoparathyroidism after total thyroidectomy (7). Transient and permanent hypocalcemia may be associated with severe symptoms, including tetany and prolonged QT interval (8), longer hospital stays, and increased hospitalization costs (9). Risk factors for postoperative hypoparathyroidism associated hypocalcemia include extent of surgery, thyroid gland size, thyroidectomy for definitive treatment of Graves' disease and for thyroid cancer, and reoperation (10–12). Surgical and medical strategies to minimize these complications include parathyroid gland autotransplantation and prophylactic administration of calcium and calcitriol (13, 14).

In an effort to identify and mitigate complications of hypoparathyroidism and hypocalcemia, several monitoring strategies are incorporated into clinical practice, such as the use of intraoperative (ioPTH) and postoperative parathyroid hormone (PTH) levels as well as the assessment of preoperative and postoperative calcium levels (15–17). In particular, low ioPTH and postoperative PTH levels correlate with early recognition of postoperative hypocalcemia, allowing for timely intervention via supplemental calcium and calcitriol administration (18, 19). Monitoring ioPTH at multiple timepoints provides the surgeon the awareness of a decline in ioPTH levels, affording an opportunity for early administration of calcitriol intraoperatively, and allows for close examination of the resected specimen for parathyroid tissue that may be salvaged via autotransplantation (20–22). The use of surgical monitoring strategies, ioPTH in particular, has primarily been studied in adults with limited data in children (23, 24).

The objective of our study was to review the clinical utility of ioPTH in preventing postoperative symptomatic hypocalcemia in pediatric and adolescent patients undergoing thyroidectomy and to assess the correlation of ioPTH levels with time of recovery for parathyroid gland function in post-operatively. We hypothesized that low ioPTH as well as a decrease in ioPTH during surgery was correlated with a longer time of recovery (TOR) from hypoparathyroidism.

## Methods

### Patients

We conducted a retrospective chart review of patients who underwent total thyroidectomy at the Children's Hospital of Philadelphia (CHOP) between December 2012 and May 2016. The study was approved by CHOP's institutional review board. Patient demographics, indication for surgery, and laboratory test values were abstracted from the electronic medical record.

Patients were included in the analysis if they had: (1) three intraoperative parathyroid hormone timepoints and (2) at least one postoperative PTH timepoint. Patients were excluded from analysis if they had incomplete laboratory data, a known diagnosis of parathyroid gland dysfunction or renal failure, or had thyroid lobectomy. Patients were also excluded based on the timing of postoperative PTH values (discussed in further detail below).

### Vitamin D/Preparation for Surgery

Prior to surgery, patients were either screened with serum 25-hydroxy vitamin D (25-OHD) and prescribed vitamin D3 (cholecalciferol) if their 25-OHD was <20 ng/mL In patients where 25-hydroxyvitamin D levels were not available, they received a single dose of 50,000 units D3 by mouth 1 week prior to surgery.

### PTH Monitoring Protocol

After induction with anesthesia, ioPTH was measured (*Turbo* Intact PTH assay, Siemens IMMULITE 1000 instrument, normal range: 10–65 pg/mL) just prior to skin incision (1st), 10 min (2nd), and 30 min (3rd) after thyroid gland removal. Baseline serum calcium (8.5–10.2 mg/dL), phosphorous (2.5–4.5 mg/dL), and magnesium (1.7–2.2 mg/dL) levels were measured with the initial ioPTH.

Patients were stratified according to risk of postoperative hypocalcemia. Patients were considered at high risk of postoperative hypocalcemia with a 2nd or 3rd ioPTH of < 10 pg/mL, moderate risk with an ioPTH level > 10 but < 20 pg/mL, and low risk with ioPTH ≥ 20 pg/mL (14). Patients in the high risk category (ioPTH <10 pg/ml) received a single one microgram dose of intravenous (IV) calcitriol (1,25-dihydroxyvitamin D3) in the operating room or shortly after arrival in the postoperative care unit. All patients had serum calcium and phosphorous levels checked every 6 h and high-risk patients were started on oral calcium and calcitriol therapy. The goal of administering IV calcitriol was to avoid delays associated with the time required to complete surgery and for patients to tolerate oral administration of medication postoperatively. Patients identified in the moderate risk category (ioPTH >10 pg/ml and <20 pg/ml) were started on oral calcium with the addition of oral calcitriol supplementation based on postoperative calcium and phosphorous levels. Patients in the low risk category (ioPTH >20 pg/ml) were started on oral calcium or calcitriol supplementation only if the postoperative calcium and phosphorous levels were abnormal (see link for protocol details: https://www.chop.edu/clinical-pathway/hypocalcemia-surveillance-total-thyroidectomy-clinical-pathway).

In the outpatient setting, serum calcium (8.9–10.4 mg/dL) and phosphorous (3.0–6.0 mg/dL up to 12 years of age then 2.5–4.5 mg/dL > 12 years of age) were measured weekly and oral supplements decreased with or without calcitriol based on normal values. An intact PTH (iPTH; normal = 10–65 pg/mL) was used to confirm normalization of the parathyroid gland function; however, over time iPTH was ordered with decreasing frequency secondary to the faster turn-around time, lower cost, and clinical reliability of serum phosphorous and calcium to predict parathyroid gland recovery (25, 26). All blood samples were processed at the closest CLIA-approved laboratory facility for the family's convenience.

### Statistical Analysis

Patient data was analyzed using R Studio Team (27). A *P* < 0.05 was considered statistically significant, and two-sided tests of hypotheses were used throughout. Continuous variables were expressed as mean ± standard deviation (SD) or median (range) for non-parametric distributions. We defined time of recovery (TOR) as the time difference between the first ioPTH timepoint and subsequent timepoint post-thyroidectomy where PTH was within the normal reference range (10–65 pg/mL).

We excluded patients when their calculated TOR required using an initial postoperative PTH timepoint >100 h to optimize the accuracy and reliability of calculating TOR. We used a linear regression to examine the relationship of TOR with variables of interest including sex, age, and ioPTH levels. Analysis of Variance (ANOVA) and Tukey Honest Significant Difference (HSD) test were performed on subgroups of patients stratified by tertile risk level.

## Results

In our analysis, 65 patients (54 female) with a median age of 15 years (range 5–23 years) were included in the analysis with the indication for surgery divided between Graves' disease (*n* = 29) and thyroid cancer (*n* = 36) (Table 1). No patient developed symptomatic hypocalcemia that required IV calcium administration as determined by our clinical practice guideline (https://www.chop.edu/clinical-pathway/hypocalcemia-surveillance-total-thyroidectomy-clinical-pathway).

**Table 1:** Demographics of patients included in the data analysis.

**Demographic variable**	**Total**
**Sex**	65
Female	54
Male	11
**Race**	
White	29
African American	5
Asian	3
Other	5
Not specified	23
**Age (years)**	
Mean	15.1
Range	5.6–22.8
**Indication for surgery**	
Graves' disease	29
Thyroid cancer	36

All patients in the study cohort achieved normalization of parathyroid gland function based on normal serum calcium and phosphorus levels off of supplement calcium and calcitriol. The average TOR for all 65 patients was 9.8 ± 34.99 days (235.5 h ± 839.84 h). Age, sex, ethnicity, and the indication for thyroidectomy (benign disease compared to thyroid cancer) were not significantly associated with TOR (data not shown).

**Figure 1:**
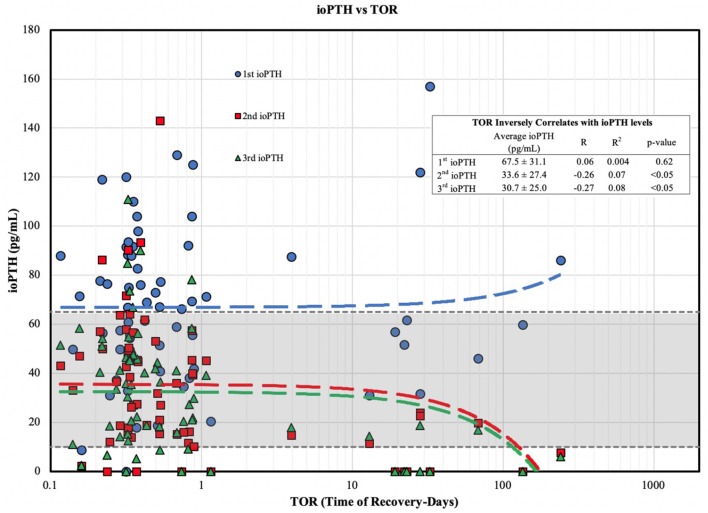
ioPTH vs. TOR for parathyroid gland function. TOR shows an inverse correlation with ioPTH levels divided into risk of post-operative hypoparathyroidism tertiles. The 2nd (red line) and 3rd (green line) ioPTH values are the most predictive for the timing of TOR (in days).

**Figure 2:**
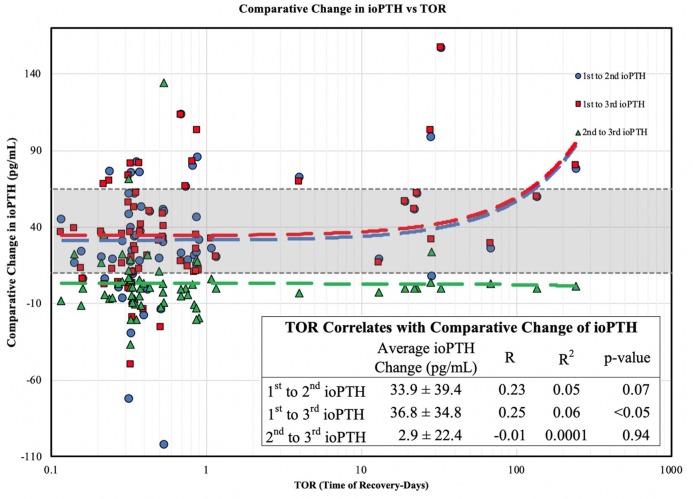
Comparative change in ioPTH vs. TOR. The change (delta) between the 1st and 3rd ioPTH correlates with parathyroid gland recovery.

When we examined the ioPTH time point to TOR we found a statistically significant inverse correlations between TOR and the 2nd (*R* = −0.26; *R*^2^ = 0.07) and 3rd (*R* = −0.27; *R*^2^ = 0.08) ioPTH levels; a longer TOR was associated with a lower 2nd and 3rd ioPTH level (Figure 1). Analyzing the TOR to the comparative change between 1st and 2nd, 2nd and 3rd, and 1st and 3rd ioPTH to TOR there was a statistically significant correlation between the 1st and 3rd ioPTH to TOR (*R* = 0.25, *R*^2^ = 0.06, *p* < 0.05) but not between 1st and 2nd or the 2nd and 3rd time points (Figure 2).

Stratifying patients into high, moderate and low risk groups, the TOR (in days) decreased by orders of magnitude from an average of 43.13 ± 76.00 days for high-risk to 6.10 ± 17.44 to 1.85 ± 6.20 days for the 2nd ioPTH and 33.62 ± 66.58 days to 8.96 ± 19.49 to 0.46 ± 0.25 days for the 3rd ioPTH (Table 2). Despite the wide variation in TOR that reflects reality of clinical practice, the mean TOR for the 2nd and 3rd ioPTH was were statistically significant. *Post-hoc* analysis between TOR and ioPTH risk groups confirmed a significant relationship between the high risk and low risk groups for the 2nd and 3rd ioPTH as well as a significant relationship between the high risk and medium risk groups for the 2nd ioPTH (Table 3).

**Table 2:** Patients with high risk 2nd and 3rd ioPTH (< 10 pg/mL) have greater parathyroid gland TOR (in days) than patients with medium and low-risk ioPTH.

	**High risk ioPTH ≤10**	**Medium risk 10 < ioPTH <20**	**Low risk ioPTH ≥ 20**	***p*-value**
1st ioPTH	0.24 ± 0.11 (2)	0.44 ± 0.10 (2)	10.43 ± 36.05 (61)	0.860
2nd ioPTH	43.13 ± 76.00 (11)	6.10 ± 17.44 (15)	1.85 ± 6.20 (39)	<0.01
3rd ioPTH	33.62 ± 66.58 (15)	8.96 ± 19.49 (13)	0.46 ± 0.25 (37)	<0.01

**Table 3:** *Post-hoc* comparison between high risk to medium risk as well as between high risk to low risk ioPTH indicates statistically significant differences in parathyroid gland TOR at the 2nd and 3rd ioPTH time points.

***p*-value**	**High risk medium risk**	**High risk low risk**	**Medium risk low risk**
1st ioPTH	0.90	0.90	0.90
2nd ioPTH	<0.05	<0.01	0.89
3rd ioPTH	0.12	<0.01	0.69

## Discussion

Total thyroidectomy is associated with transient hypoparathyroidism in up to 20% of patients and permanent hypoparathyroidism in up to 12% of pediatric patients (1, 5). Our data supports the clinical utility of using ioPTH to identify patients at increased risk of hypoparathyroidism and our clinical practice guideline has decreased our rate of symptomatic, postoperative hypocalcemia (7). Low ioPTH, as well as the extent of decrease in ioPTH, correlates with a longer TOR from postoperative hypoparathyroidism. Importantly, we found there is a significant difference in the time for medium (ioPTH > 10 and <20 pg/mL) and high risk (ioPTH <10 pg/mL) patients to reach normal parathyroid hormone levels. Finally, we found no significant linear association between TOR and demographic variables such as age, sex, ethnicity, or the indication for surgery. While several previous studies have reported an association with younger age at the time of surgery, Graves' disease, and lymphadenectomy (28, 29), the lack of association between TOR and surgical diagnosis in this study may be biased by the overall low rate of permanent hypoparathyroidism for our thyroid surgeons (7). A recent report of 106 children undergoing total thyroidectomy reports a similar lack of association between hypoparathyroidism and surgical indication (30).

Our findings demonstrate the usefulness in obtaining intraoperative PTH levels to predict the risk of postoperative hypocalcemia as well as the timing for parathyroid gland recovery, a critical factor to for anticipatory guidance in regard to optimizing the timing and frequency of postoperative laboratory surveillance. Previous studies have recommended that obtaining an ioPTH at the end of surgery is the most informative to identify patients that benefit from calcitriol and calcium supplementation (25, 31). The finding of an elevated initial ioPTH post-induction and pre-incision is likely secondary to an anesthetic effect (32). Although the mechanism for this transient rise in ioPTH is not well-defined, the proposed etiology is an acute and transient anesthesia induced decrease in ionized and total calcium and albumin leading to an increase in ioPTH (33). In our cohort, the initial ioPTH does not appear to be related to vitamin D deficiency as all of our patients either received a single dose of 50,000 units D3 by mouth 1 week prior to surgery or were evaluated for vitamin D deficiency and treated if 25-OHD was <20 ng/mL. This assumption is supported by data showing a delta increase in 25-OHD of approximately 30 ng/ml 3 days after administration of 50,000 IU of either vitamin D2 or D3 with maintenance of 25-OHD levels for 7 to 14 days (34).

Since parathyroid gland recovery correlates with intraoperative PTH at the 2nd and 3rd time points of measurement (Figure 1), these two time points are the most informative to help guide both short and long-term postoperative surveillance and management. Medium risk (ioPTH between 10 and 20 pg/mL) and high-risk patients (ioPTH <10 pg/mL) should be placed on calcium supplementation (medium risk) with calcitriol (high risk), and patients should be informed that their parathyroid gland recovery could take 3–4 months (Table 1). In particular, physicians should follow these patients more closely to verify a full recovery and ensure that they have sufficient medication (calcium and vitamin D supplements) as well as regular laboratory testing. In contrast, low risk patients (ioPTH > 20) are far less likely to have prolonged recovery times. The cut-off levels for the ioPTH tertiles in the CHOP clinical practice guideline used to define the risk of postoperative hypocalcemia are associated with a reduced incidence of symptomatic hypocalcemia (7) and supported by several recent studies showing an increased risk of postoperative hypoparathyroidism for ioPTH <11 pg/ml (31, 35), and a lower risk for ioPTH > 16 pg/ml (24).

Incorporating ioPTH measurement into surgical care allows for identification of patients at risk of postoperative hypocalcemia at the earliest intraoperative time point and provides the surgeon the opportunity for identifying devascularized parathyroid gland(s) that may be salvaged through autotransplantation. In addition, the early identification of patients at risk for postoperative hypocalcemia has allowed for early initiation of supplemental calcium and calcitriol. With the initiation of our post-thyroidectomy management protocol using the ioPTH tertile risk levels to identify patients at risk of hypocalcemia there have been no patients in our practice that have required IV calcium in the intraoperative period between 2014 and 2017 (7) with only one patient requiring IV calcium for symptomatic hypocalcemia between 2017 and 2019 (unpublished). Lastly, while there was a broad standard deviation in the timing of parathyroid gland recovery (TOR; Table 1), the mean and median values have been clinically useful to predict, direct surveillance, and provide anticipatory guidance in regard to the potential delayed recovery of parathyroid gland function based on ioPTH risk tertile.

This study has limitations. First, our study was unable to predict and correlate permanent hypoparathyroidism since all patients in this study experienced full recovery of parathyroid gland function. Second, due to our strict inclusion/exclusion criteria, and the general practice of only following calcium and phosphorous levels during outpatient surveillance of parathyroid gland recovery, our study was limited to only 65 patients. A prospective study with a larger cohort may help determine the generalizability of our findings. Finally, our calculation of TOR is constrained by the frequency and timing of our patient's laboratory testing. As such, there is a large standard deviation in the tertile TORs with an inability to provide an exact time and a greater variance with respect to our patient's exact timing of parathyroid gland recovery. Nonetheless, in our practice, the TOR range is a clinically proven, useful guide for patient care.

## Conclusion

This study demonstrates the usefulness of ioPTH levels to predict and prevent symptomatic postoperative hypocalcemia as well as the timing of parathyroid gland recovery in pediatric patients undergoing total thyroidectomy. The 2nd and 3rd ioPTH levels are most predictive of postoperative hypoparathyroidism, and also predict the timing of parathyroid gland recovery. Incorporation of ioPTH into a clinical practice guideline can optimize the clinical utility of ioPTH and provides an opportunity to individually customize post-thyroidectomy outpatient calcium management and laboratory surveillance. It can also be used to provide the patient and family with appropriate anticipatory guidance in regard to the timing of parathyroid gland recovery.

## Data Availability Statement

The datasets used and/or analyzed during current study are available from the corresponding author upon reasonable request.

## Ethics Statement

The studies involving human participants were reviewed and approved by Children's Hospital of Philadelphia Institutional Review Board. Written informed consent from the participants' legal guardian/next of kin was not required to participate in this study in accordance with the national legislation and the institutional requirements.

## Author Contributions

AB: conception and design and provision of study material or patients. ST, SB, and AB: collection and assemble of data and data analysis and interpretation. All authors: manuscript writing and final approval of all manuscript.

### Conflict of Interest

AB received a lecture honorarium from Sandoz AG. The remaining authors declare that the research was conducted in the absence of any commercial or financial relationships that could be construed as a potential conflict of interest.

## Abbreviations

ANOVA: Analysis of Variance
CHOP: Children's Hospital of Philadelphia
HSD: Tukey Honest Significant Difference Test
ioPTH: Intraoperative PTH
PTH: Parathyroid Hormone
TOR: Time of Recovery.
